# Etiological characteristics and genotypes of *Mycoplasma pneumoniae* infection in children in Baoding from 2022 to 2024

**DOI:** 10.1128/spectrum.01089-26

**Published:** 2026-09-18

**Authors:** Ze-ming Wang, Ting-ting Jiang, Wei-wei Jiao, Tian-yi Wang, Lu Yu, Jing-jing Chu, Kai-xuan Cao, Yu-ping Chen, Cai-xia Liu, Jie Tan, Alexander V. Dmitriev, Jing Bi, A-dong Shen

**Affiliations:** 1 Department of Pediatrics, Beijing Friendship Hospital, Capital Medical University 12517, Beijing, People's Republic of China; 2 Baoding Key Laboratory for Precision Diagnosis and Treatment of Infectious Diseases in Children, Hebei Key laboratory of Infectious Diseases Pathogenesis and Precise Diagnosis and Treatment, Baoding Hospital of Beijing Children’s Hospital, Capital Medical University, Baoding, Hebei Province, People's Republic of China; 3 Beijing Key Laboratory of Pediatric Respiratory Infection Diseases, Key Laboratory of Major Diseases in Children, Ministry of Education, National Clinical Research Center for Respiratory Diseases, Laboratory of Respiratory Diseases, National Center for Children’s Health, Beijing Pediatric Research Institute, Beijing Children’s Hospital, Capital Medical University 12517, Beijing, People's Republic of China; 4 Department of Molecular Biotechnology, Saint-Petersburg State Institute of Technology 387222, Saint-Petersburg, Russia; University of South Florida, Tampa, Florida, USA

**Keywords:** *Mycoplasma pneumoniae*, pneumonia, macrolide resistance, genotype, epidemiology, post-pandemic

## Abstract

To investigate the epidemiology associated with genetic types and macrolide resistance of *Mycoplasma pneumoniae* (MP) in children during and post-COVID-19 in Baoding. A retrospective analysis was conducted on 13,534 children and adolescents (≤18 years old) with community-acquired pneumonia from January 2022 to December 2024. MP detection and macrolide resistance-associated mutation analysis were performed using multiplex real-time PCR. Multilocus variable-number tandem-repeat analysis (MLVA) was used for genotyping. *Mycoplasma pneumoniae* pneumonia (MPP) cases rose significantly from 844 (2022) to 6,346 (2024) (*P* < 0.01), while the positive rate remained stable (67.68%–77.72%). Patients aged 5–18 years had the highest positive rate. Severe MPP (SMPP) prevalence declined from 22.75% (2022) to 6.16% (2024) (*P* < 0.01), with MP infections peaking in the fourth quarter of 2023–2024 and prevailing year-round in 2024. Macrolide-resistant MP (MRMP) prevalence was 95.92% overall (97.27%/2022, 93.85%/2023, 96.56%/2024), with no significant difference between SMPP and non-severe MPP groups. MLVA-type M3562 decreased from 77.10% to 33.13%, while M4572 increased to 56.72% (2024) as the dominant type. Both genotypes had 99.00%–100.00% macrolide resistance. Notably, SMPP was more common in M3562-infected patients (20.63%) than M4572-infected ones (13.97%, *P* < 0.01). MP infections re-emerged between 2023 and 2024, with persistently high macrolide resistance. There was a genotype shift from M3562 to M4572. These findings highlight the need for strengthened surveillance, rational antibiotic use, and targeted prevention strategies to address MP infections in the post-pandemic period.

**IMPORTANCE:**

Understanding the epidemiological characteristics of *Mycoplasma pneumoniae* (MP) is essential for effective management of *Mycoplasma pneumoniae* pneumonia (MPP). This study examines the epidemiology associated with genetic types and macrolide resistance of MP in children with MPP in Baoding, China, during and after the lifting of non-pharmaceutical interventions (NPIs). Data from 9,728 children diagnosed with MPP from January 2022 to December 2024 were analyzed. MPP cases rose significantly from 844 in 2022 to 6,346 in 2024, while the overall prevalence of macrolide-resistant MP was 95.92%. Notably, a striking genotype shift from M3562 (77.10%/2022–33.13%/2024) to M4572 (15.74%/2022–56.72%/2024) was observed over this period. Severe MPP proportion was significantly higher in patients with M3562 (20.63%) infection than that with M4572 (13.97%). This research underscores the urgent need for strengthened surveillance, rational antibiotic use, and targeted prevention strategies to address MP infections in the post-pandemic period.

## INTRODUCTION

*Mycoplasma pneumoniae* (MP) remains a leading cause of community-acquired pneumonia (CAP) among children and adolescents (1). MP can cause severe pneumonia or extrapulmonary manifestations requiring hospitalization and is responsible for 10%–40% of CAP cases in children (2). MP infections occur globally as both endemic and epidemic outbreaks, with epidemic peaks every 1–7 years (3–5). The prolonged incubation period (up to 3 weeks) and relatively low transmission rate contribute to the lengthy duration of MP epidemics (6), while recurrent outbreaks are thought to stem from declining herd immunity or the emergence of new subtypes within the population (3, 5, 6). Notably, the latest MP epidemic emerged from early 2023 to 2024 following the relaxation of non-pharmaceutical interventions (NPIs) post-COVID-19, with mean detection rates being 10.30% in Europe, 19.66% in Asia, 1.34% in the Americas, and 0.56% in Oceania (7), while the detection rates reached 50%–70% in China (8–11), at least 3 to 5 times higher than pre-pandemic levels (11).

Macrolides are the first-line antibiotics for the treatment of *Mycoplasma pneumoniae* pneumonia (MPP) in children (12). However, since the 2000s, the prevalence of macrolide-resistant MP (MRMP) has risen rapidly, becoming a severe and intractable challenge in the management of childhood MPP worldwide, particularly in China (13). Studies have demonstrated significant regional and temporal variations in the macrolide resistance rate of MP, which may be associated with the widespread use of antibiotics and evolutionary changes in MP genotypes (7, 10).

Molecular typing is essential for understanding the epidemiological dynamics and evolutionary trends of MP. Three major typing methods have been developed for MP surveillance, each targeting different genomic regions and offering distinct resolution. Multilocus variable-number tandem-repeat analysis (MLVA) targets four variable-number tandem repeat (VNTR) loci (Mpn13–16) and provides high discriminatory power at the sub-lineage level, making it well-suited for outbreak investigations and short-term epidemiological monitoring. P1 typing, based on sequence variations in the p1 gene encoding the major adhesin and immunogen, classifies strains into two main types (P1-1 and P1-2) and their variants. As the primary mediator of cytoadherence and a dominant target of the host immune response, P1 is directly relevant to pathogenesis, and P1 type shifts may influence population-level immunity and epidemic dynamics. Multilocus sequence typing (MLST) targets housekeeping genes and yields portable, highly reproducible sequence types (STs) suitable for global phylogenetic comparisons. These three methods are complementary rather than interchangeable: MLVA types have been shown to correlate with P1 types, with MLVA clusters generally nesting within P1 type groupings (14–17), while MLST provides broader phylogenetic context at the clonal complex level.

The clinical and epidemiological relevance of P1 typing is further underscored by its association with macrolide resistance. Most of the type P1-1 strains recently isolated in Asian countries were MRMP, whereas type P1-2 strains were mostly macrolide-susceptible *M. pneumoniae* (17). Epidemiological data from 18 surveillance sites show that MP’s macrolide resistance rate has remained consistently low in European countries, such as France, Belgium, England, Denmark, and Slovenia, associated with ceftriaxone most commonly prescribed to hospitalized children in Europe rather than azithromycin in China (18, 19), with distinct subtype distribution in Europe (M3562 and P1-2 with low drug resistance), low transmission rates of MR-MP strains, and the absence of comprehensive national surveillance systems in most European countries (20, 21). In contrast, an upward trend has been observed in Asian regions, including China, South Korea, and Taiwan of China (7, 10).

Since the COVID-19 pandemic, global NPIs have significantly altered the transmission dynamics of respiratory pathogens, including MP (7, 22). Following NPIs relaxation, MP infections have resurged in many regions, with changes in epidemic intensity, seasonality, and clinical severity compared to the pre-pandemic period (7, 11, 23). However, differences in MP epidemic characteristics, particularly MRMP prevalence and genotype distribution after post-NPIs, remain limited due to insufficient long-term monitoring.

This study investigated MP infections in children and adolescents with MPP from January 2022 to December 2024. We analyzed temporal trends, seasonality, disease severity of MPP, MRMP prevalence, and MP genotype distribution via MLVA. We also explored correlations between genotypes, macrolide resistance, and clinical severity. These findings provide critical epidemiological data to inform the clinical management, prevention, and control of MPP in the post-pandemic era.

## MATERIALS AND METHODS

### Study population and specimen collection

A retrospective analysis was conducted on clinical data from children aged ≤18 years who were diagnosed with CAP and required hospitalization at Baoding Hospital of Beijing Children’s Hospital Affiliated to Capital Medical University, a regional medical center serving Baoding and its surrounding areas, from 1 January 2022 to 31 December 2024. The diagnosis of CAP was based on signs and symptoms of acute respiratory infection and chest radiograph findings of suspected MPP (consolidation, infiltration, or interstitial changes) consistent with established national guidelines for pediatric MPP (12).

In this study, patients with MPP enrolled met the criteria of CAP and a positive polymerase chain reaction (PCR) result. The children with MPP were categorized into severe MPP (SMPP) and non-severe MPP (NSMPP) groups according to clinical manifestations, in adherence to the aforementioned guidelines (12, 24). SMPP was defined as MPP complicated by one or more of the following criteria: (i) poor general condition (pale or gray complexion and inadequate response to the surrounding environment); (ii) impaired consciousness; (iii) cyanosis; respiratory rate >70 breaths/min in infants or >50 breaths/min in children aged >1 year; assisted breathing (including groaning, nasal flaring, and triple concavity sign); intermittent apnea; or oxygen saturation <92%; (iv) ultra-high fever (≥41°C) or persistent high fever (≥39°C) lasting >5 days; (v) signs of dehydration or refusal to eat; (vi) radiological findings, including pulmonary infiltration, involving ≥2/3 of one lung, multilobar infiltration, pleural effusion, pneumothorax, atelectasis, pulmonary necrosis, or lung abscess; (vii) extrapulmonary complications during the hospitalization.

Exclusion criteria included children with recent hospitalization within the previous 15 days; clear alternative diagnoses (e.g., asthma, upper respiratory infections, respiratory tumors, cystic fibrosis, congenital metabolic disorders, primary ciliary dyskinesia, immunodeficiency disorders), receipt of immunosuppressive therapy, recent solid-organ or hematopoietic stem-cell transplant within the past 90 days, duplicate detections; and incomplete clinical records.

Appropriate respiratory specimens (sputum, throat swab, or bronchoalveolar lavage fluid) were collected from children with CAP upon hospitalization. Specimens were transferred to the qualified laboratory at Baoding Hospital of Beijing Children’s Hospital and tested within 24 h, or stored at −80°C until analysis. Demographic data and clinical characteristics were extracted from the hospital’s electronic medical records.

### Detection of *M. pneumoniae* and macrolide resistance-associated mutations

MP and macrolide resistance-associated mutations were detected using an MP Detection Kit (Mole, Jiangsu, China) assay with a detection limit of 500 copies/mL (25). The P1 gene was targeted to confirm MP presence with a probe labeled with VIC, while two point mutations (A2063G and A2064G) in domain V of the 23S rRNA gene were analyzed to determine macrolide resistance with a probe labeled with FAM. Samples were confirmed as MRMP if both VIC and FAM fluorescence signals were detected. MP-positive DNA remnants were stored at −80°C for subsequent MLVA typing.

### MLVA typing

MLVA genotyping was performed targeting four VNTR loci (Mpn13, Mpn14, Mpn15, and Mpn16) via nested PCR with previously published primers (26, 27). PCR products were sequenced, and VNTR copy numbers were calculated to define MLVA types (28).

### Statistical analysis

Categorical data were analyzed using the chi-square test or Fisher’s exact test, and continuous data were analyzed using Student’s *t*-test. SPSS software version 25.0 was used for statistical analysis, with a *P* value < 0.05 considered statistically significant.

## RESULTS

### Outbreak of MP epidemics in Baoding following the full lifting of COVID-19 restrictions

From January 2022 to December 2024, 13,534 hospitalized participants with CAP were enrolled after applying exclusion criteria, and 9,728 (71.88%) were diagnosed with MPP.

**Fig 1:**
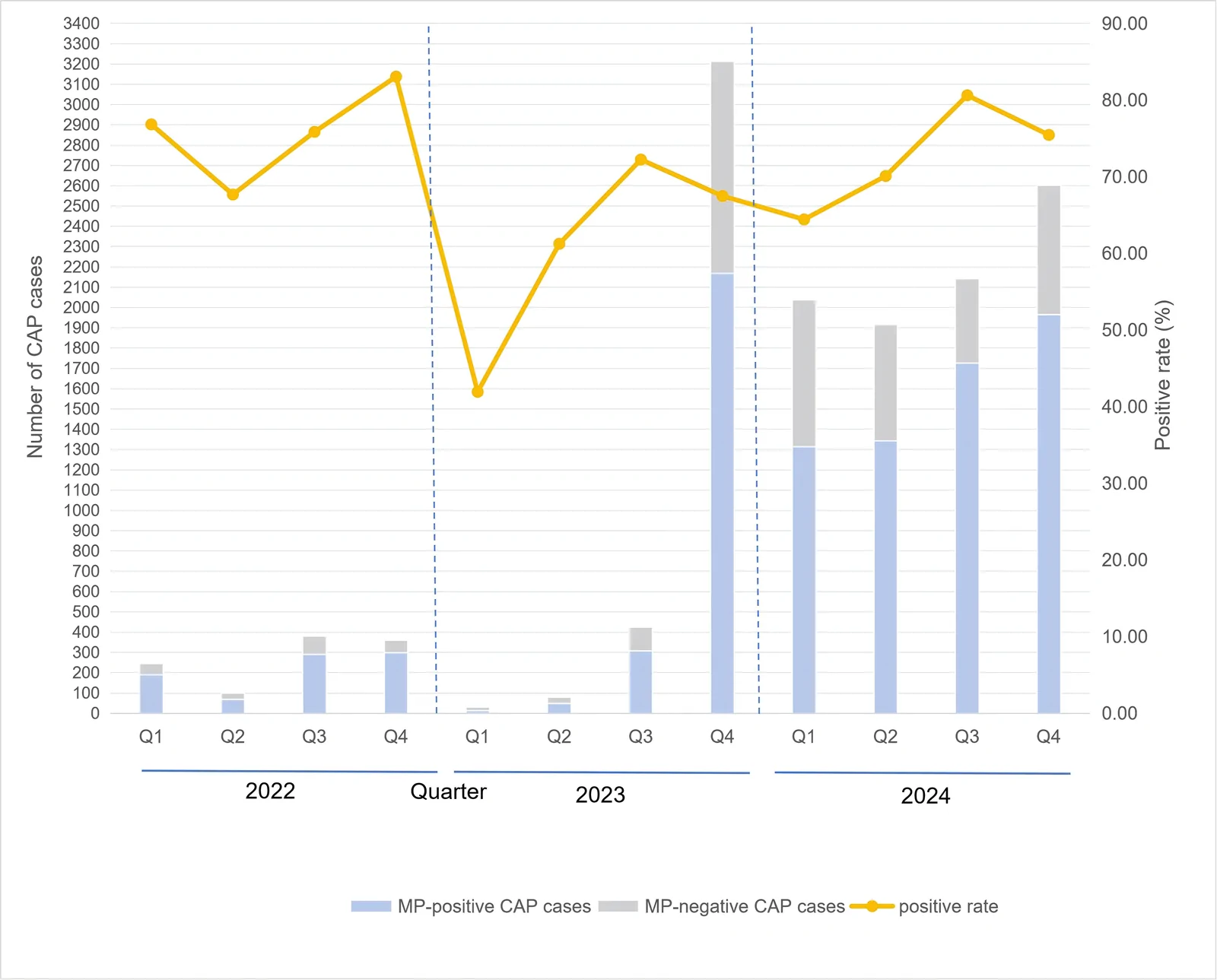
MP-positive cases and rates from 2022 to 2024: the bar illustrated the number of positive (blue) or negative (gray) cases observed in each quarter, while the dotted line represented the positive rate of MP.

Over the study period, the total number of MP-positive cases showed a significant upward trend (*P* < 0.01), increasing from 844 in 2022 to 2,538 in 2023 and peaking at 6,346 in 2024. However, the overall MP positive rate remained relatively stable, with 77.72% in 2022, 67.68% in 2023, and 72.96% in 2024. Among MP-positive patients, males accounted for 51.54%, with a median age of 7 years (interquartile range, 5–9 years). Children aged 5–18 years consistently demonstrated the highest positivity rate between 2022 and 2024. A total of 970 children (9.97%) were diagnosed with SMPP, with a gradual decline in prevalence from 22.75% in 2022 to 6.16% in 2024 (*P* < 0.01) (Table 1; Fig. 1).

**TABLE 1:** Demographic characteristics of recruited patients with MPP from 2022 to 2024^*a*^

Variables	2022 (*N* = 844)	2023 (*N* = 2,538)	2024 (*N* = 6,346)	Total (*N* = 9,728)	χ2	*P* value
Sex						
Male	444 (52.61)	1,266 (49.88)	3,304 (52.06)	5,014 (51.54)	2.877	0.144
Female	400 (47.39)	1,272 (50.12)	3,042 (47.94)	4,714 (48.46)		
Age group						
0–1 year	49 (5.81)	90 (3.55)	289 (4.55)	428 (4.40)	136.797	<0.01
2–4 years	160 (18.96)	290 (11.43)	1,353 (21.32)	1,803 (18.53)		
5–9 years	487 (57.70)	1,652 (65.09)	3,697 (58.26)	5,836 (59.99)		
10–18 years	148 (17.54)	506 (19.94)	1,007 (15.87)	1,661 (17.07)		
Quarter of infection						
Q1 (January–March)	189 (22.39)	13 (0.51)	1,313 (20.69)	1,515 (15.57)	2,397.588	<0.01
Q2 (April–June)	67 (7.94)	49 (1.93)	1,343 (21.16)	1,459 (15.00)		
Q3 (July–September)	289 (34.24)	307 (12.10)	1,726 (27.20)	2,322 (23.87)		
Q4 (October–December)	299 (35.43)	2,169 (85.46)	1,964 (30.95)	4,432 (45.56)		
Sample type						
Throat swabs	0	0	4 (0.06)	4 (0.04)	552.504	<0.01
Sputum	707 (83.77)	2,457 (96.81)	6,258 (98.61)	9,422 (96.85)		
Bronchoalveolar lavage fluid	137 (16.23)	81 (3.19)	84 (1.32)	302 (3.10)		
Severity						
SMPP	192 (22.75)	387 (15.25)	391 (6.16)	970 (9.97)	334.840	<0.01
NSMPP	652 (77.25)	2,151 (84.75)	5,955 (93.84)	8,758 (90.03)		

^*a*^ SMPP, severe *Mycoplasma pneumoniae* pneumonia; NSMPP, non-severe *Mycoplasma pneumoniae* pneumonia; data are no. (%). The *P *values were calculated to compare the overall distribution of each variable across years.

As shown in Fig. 1, MP infections and positive rates exhibited distinct quarterly variation (*P* < 0.01). Following the relaxation of COVID-19 restrictions in late 2022, MP infections peaked in the fourth quarter (October–December) of both 2023 and 2024. Additionally, MP infections prevailed throughout 2024, accounting for 65.23% of 3-year total MPP cases during 2022 and 2024.

### Persistently high macrolide resistance in epidemic MPP in Baoding after NPIs

Among the 9,728 patients with MPP, a substantial 9,331 cases (95.92%) were identified as MRMP. The MRMP rates remained alarmingly high throughout 2022 to 2024, reaching 97.27% in 2022, 93.85% in 2023, and 96.56% in 2024. MRMP rates were significantly high in both SMPP and NSMPP groups, with no significant difference between the two populations (Table 2).

**TABLE 2:** Characteristics of patients with MRMP from 2022 to 2024^*a*^

Variables	Cases (*N*)	Proportion (%)	Resistance rate (%)
Age group			
0–1 year	401	4.30	93.69
2–4 years	1,713	18.36	95.01
5–9 years	5,618	60.21	96.26
10–18 years	1,599	17.14	96.27
Year			
2022	821	8.80	97.27
2023	2,382	25.53	93.85
2024	6,128	65.67	96.56
Quarter of infection			
Q1 (January–March)	1,466	15.71	96.77
Q2 (April–June)	1,429	15.31	97.94
Q3 (July–September)	2,268	24.31	97.67
Q4 (October–December)	4,168	44.67	94.04
Severity			
SMPP	941	10.08	97.01
NSMPP	8,390	89.92	95.80

^*a*^ SMPP, severe *Mycoplasma pneumoniae* pneumonia; NSMPP, non-severe *Mycoplasma pneumoniae* pneumonia.

### Significant genotype conversion from M3562 to M4572 and a higher SMPP proportion in M3562 infection in Baoding after COVID-19

**Fig 2:**
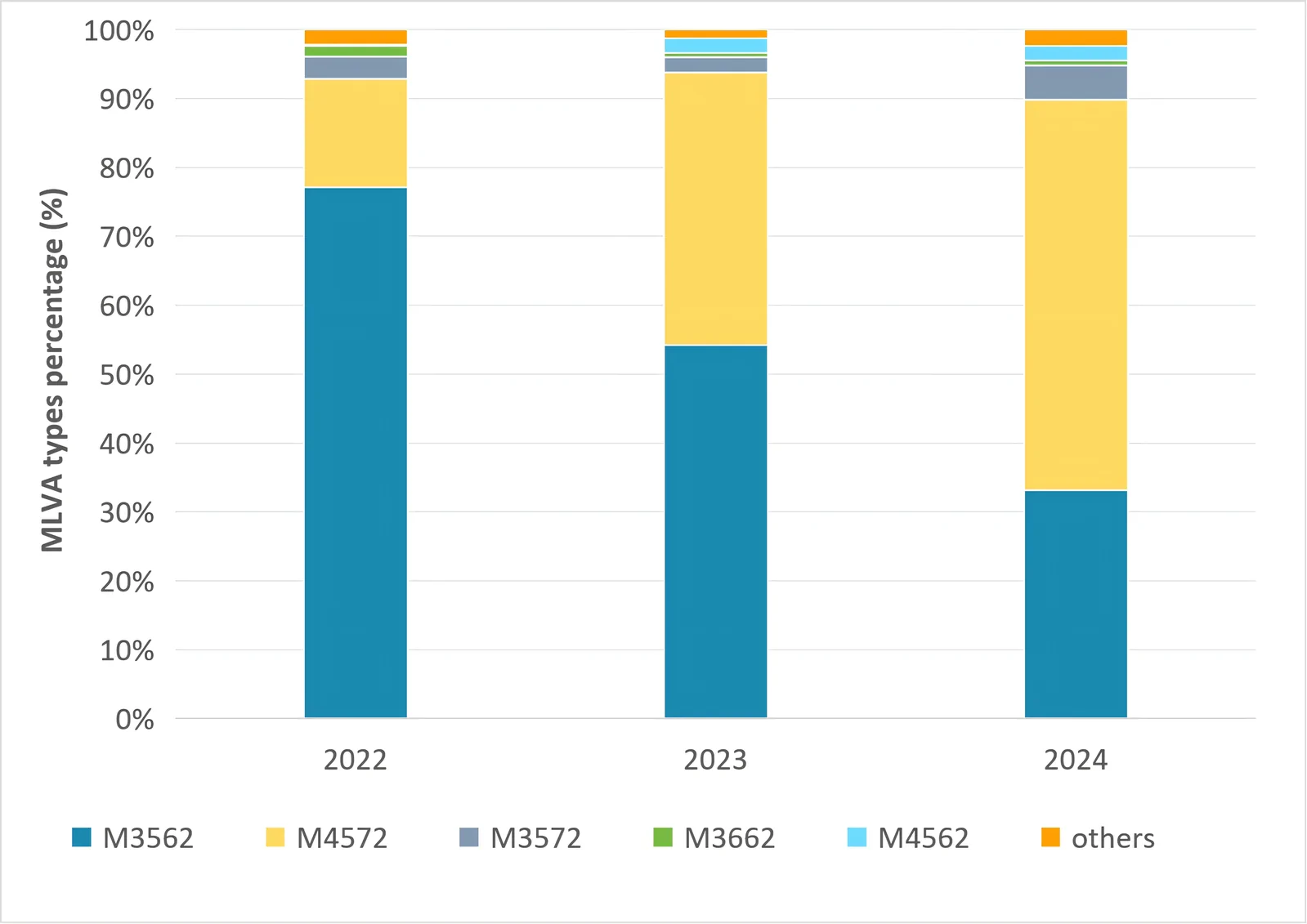
Distribution of different MLVA types of MP from 2022 to 2024 (*N* = 4,281).

MLVA genotypes were successfully identified in 4,281 patients, revealing 22 types. The M3562 (42.79%) and M4572 (48.17%) genotypes were predominant and consistently present throughout the study period. The proportion of the M3562 genotype showed a continuous downward trend, decreasing from 77.10% in 2022 to 54.20% in 2023 and further to 33.13% in 2024. In contrast, the M4572 genotype exhibited a marked upward trend, rising from 15.74% in 2022 to 39.52% in 2023 and becoming the most prevalent genotype in 2024 (56.72%). Other rare genotypes accounted for only 9.04% of the total from 2022 to 2024 (Fig. 2).

**Fig 3:**
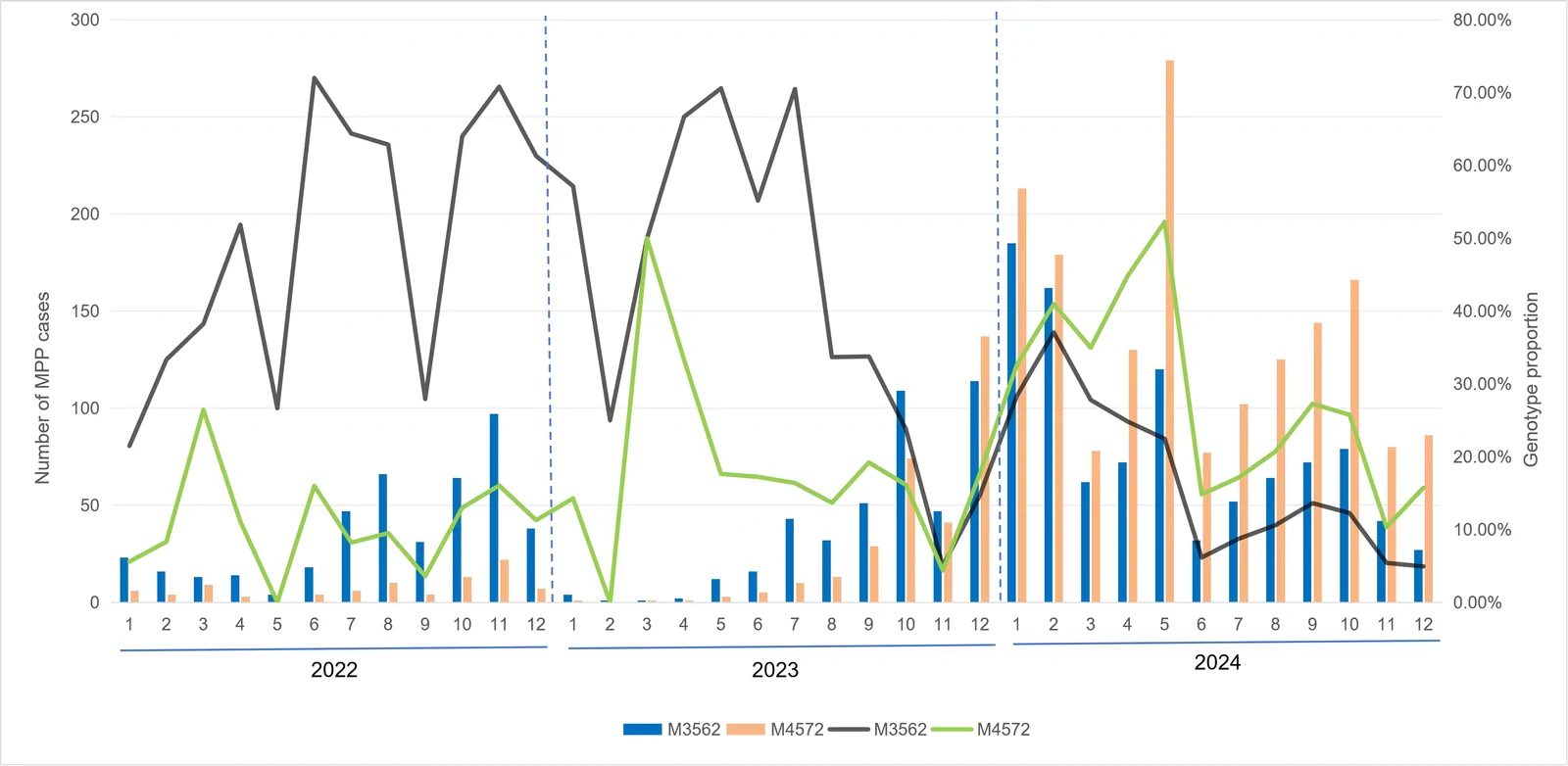
MP genotype cases and proportion each month from 2022 to 2024: the line represented the proportion of M3562 (black) or M4572 (green) each month, while the bar illustrated the number of M3562 (blue) or M4572 (orange) cases observed each month.

Further epidemiological analysis of the M3562 and M4572 genotypes demonstrated that before November 2023, the prevalence of M3562 consistently surpassed that of M4572. Significantly, during November 2023—coinciding with a substantial surge in MPP cases—the proportions of these two genotypes converged. Subsequently, the proportion of M4572 exceeded that of M3562 after December 2023 (Fig. 3).

As shown in Table 3, the two predominant MLVA genotypes (M4572 and M3562) were significantly more prevalent in the MRMP group than in the macrolide-sensitive MP group (99.85% vs 0.15% and 99.89% vs 0.11%, respectively; *P* < 0.01). When stratified by year, the macrolide resistance rates of both genotypes remained persistently high (99.00%–100.00%).

**TABLE 3:** MLVA types and macrolide resistance

MLVA types (*n*, %)	Macrolide resistance (%)	
	Resistant (*n* = 4,275)	Sensitive (*n* = 6)
M4572 (2,062, 48.17)	2,059/2,062 (99.85%)	3/2,062 (0.15%)
M3562 (1,832, 42.79)	1,830/1,832 (99.89%)	2/1,832 (0.11%)
M3572 (180, 4.20)	179/180 (99.44%)	1/180 (0.56%)
M4562 (78, 1.82)	78/78 (100.00%)	0 (0.00%)
M3662 (37, 0.86)	37/37 (100.00%)	0 (0.00%)
M4672 (28, 0.65)	28/28 (100.00%)	0 (0.00%)
M5572 (14, 0.33)	14/14 (100.00%)	0 (0.00%)
M4472 (11, 0.26)	11/11 (100.00%)	0 (0.00%)
M3582 (9, 0.21)	9/9 (100.00%)	0 (0.00%)
M4582 (9, 0.21)	9/9 (100.00%)	0 (0.00%)
M502 (4, 0.09)	4/4 (100.00%)	0 (0.00%)
M3552 (4, 0.09)	4/4 (100.00%)	0 (0.00%)
M3462 (2, 0.05)	2/2 (100.00%)	0 (0.00%)
M3472 (2, 0.05)	2/2 (100.00%)	0 (0.00%)
M5562 (2, 0.05)	2/2 (100.00%)	0 (0.00%)
M3502 (1, 0.02)	1/1 (100.00%)	0 (0.00%)
M3592 (1, 0.02)	1/1 (100.00%)	0 (0.00%)
M3762 (1, 0.02)	1/1 (100.00%)	0 (0.00%)
M4462 (1, 0.02)	1/1 (100.00%)	0 (0.00%)
M4552 (1, 0.02)	1/1 (100.00%)	0 (0.00%)
M4573 (1, 0.02)	1/1 (100.00%)	0 (0.00%)
M4662 (1, 0.02)	1/1 (100.00%)	0 (0.00%)

Further analysis of the correlation between the two predominant genotypes and disease severity revealed a markedly higher proportion of SMPP in patients infected with the M3562 genotype (20.63%) compared to those with the M4572 genotype (13.97%, *P* < 0.01) during 2022 and 2024.

## DISCUSSION

This study systematically analyzed the epidemiological characteristics, macrolide resistance patterns, and genotype distribution of MPP in children and adolescents in the Baoding area from 2022 to 2024. Key findings included a significant upward trend in MP-positive cases, persistently high MRMP prevalence, a distinct shift in dominant MLVA genotypes, and a correlation between specific genotypes and disease severity. These results provide critical insights into post-pandemic MP epidemiology and offer valuable implications for clinical management and prevention strategies.

A notable finding was the marked increase in MPP cases over the 3-year study period, rising from 844 in 2022 to 6,346 in 2024, with a surge from July 2023, despite a relatively stable overall positive rate (71.88%). This discrepancy suggests that the surge in case numbers may be primarily driven by enhanced transmission dynamics rather than changes in the proportion of infections among CAP cases. Besides MP, it was observed in other respiratory pathogens post-COVID-19 after the relaxation of NPIs in late 2022. NPIs inhibited natural pathogen circulation and prolonged low exposure to common pathogens during the pandemic, leading to decreased population immunity and increased susceptibility to subsequent infections (22, 29–32). Previous epidemiological reports have documented a marked rise in MP positivity starting in 2023. The divergence in MP positive rates observed between our investigation and prior studies can be largely explained by discrepancies in statistical denominators. In the present work, the MP positive rate was calculated based on all patients clinically suspected of MPP, whereas many other studies adopt all CAP patients as the total population for rate calculation. The stable positive rate (67.68%–77.72%) indicates that the clinical criteria for MPP remained consistent throughout the study, ensuring the reliability of cross-year comparative analyses.

*Mycoplasma pneumoniae* infections show a seasonal variation. Seasonal analysis revealed that MP infections peaked in Q4 (October–December) of 2023 and 2024, with year-round prevalence in 2024. Traditionally, MP infections exhibit seasonal fluctuations, often peaking in autumn and winter (3, 33, 34). The post-NPI resurgence appears to have retained this seasonal pattern while extending the epidemic period, possibly due to accumulated population susceptibility during NPIs and sustained transmission across seasons. This highlights the need for targeted prevention measures during peak seasons, particularly in school and community settings.

Our data showed an extremely high and sustained prevalence of MRMP (95.92%), consistent with high macrolide resistance rates reported in other Asian regions (e.g., South Korea and Taiwan of China) but in stark contrast to the low resistance rates (<10%) in European countries (7, 10, 23, 35). The widespread use of macrolides as first-line agents for childhood respiratory infections is widely recognized as the primary driver of this resistance (35, 36). Notably, MRMP rates were similarly high in both SMPP and NSMPP groups, indicating that macrolide resistance is not inherently linked to disease severity but may still impact treatment outcomes by prolonging symptom duration or increasing the risk of treatment failure (10, 37–39).

Given the established MLVA-P1 correlation, shifts in predominant MLVA types within a population may reflect underlying genetic type transitions, which in turn could signal changes in the prevalence of macrolide resistance (14, 16, 17). This genotype-resistance linkage highlights the importance of integrating molecular typing into routine surveillance. Indeed, the global distribution of MP genotypes exhibits distinct geographic patterns: P1-1 and its corresponding MLVA type M4572 are most common in the Western Pacific region, whereas P1-2 is more prevalent in the Americas, and M3562 and M3662 are predominantly found in Europe and the Americas (14, 17, 20). Beyond these geographic differences, P1 and MLVA type distribution is also highly dynamic over time. In Japan, a transition from P1-1 to P1-2 predominance occurred between 2006 and 2019 (17). In China, Beijing, Shenyang, and Baotou were predominantly affected by P1-1 strains during 2017–2019, although a rising trend of P1-2 was concurrently observed (40). By 2023, Beijing and Suzhou still exhibited a dominance of P1-1, corresponding to the MLVA type M4572 (11, 41). However, our study revealed that Baoding was primarily infected by M3562 (P1-2) in 2022, and by 2024 the predominant strain had shifted to M4572 (P1-1). This transition is consistent with the well-documented epidemiological pattern that type shifts between the two P1 lineages have occurred repeatedly at approximately 10-year intervals over the past 40 years (14, 17, 20), reflecting the inherent temporal and geographic variability of MP genotype distribution.

Remarkably, both M4572 and M3562 were strongly associated with macrolide resistance rates of 99.85% and 99.89%, respectively, while other rare genotypes also exhibited high resistance rates, further underscoring the challenges of macrolide therapy for MPP in this region. These findings indicate that macrolide resistance is widespread across major MLVA genotypes in this region, likely due to clonal expansion of resistant strains (7, 11, 42, 43). Clinicians should exercise caution when relying solely on macrolides for empirical treatment of MPP, especially in areas with high MRMP prevalence. In response to this trend, the National Health Commission of the People’s Republic of China updated the Guidelines for the Management of Pediatric Mycoplasma pneumoniae Pneumonia in 2025 (44). The revised guideline recommends that pediatric patients with a clinical diagnosis of MPP or macrolide-resistant MPP, especially those presenting with SMPP, should promptly switch to tetracyclines or fluoroquinolones (age-permitted) rather than continuing macrolide monotherapy, to improve therapeutic efficacy and reduce disease progression risks.

This study identified a striking shift in dominant MP genotypes: M3562, which accounted for 77.10% of cases in 2022, declined to 33.13% in 2024, while M4572 increased from 15.74% to 56.72% over the same period, becoming the most prevalent genotype. It may be attributed to antibiotic selective pressure, changes in host immunity, or enhanced transmission fitness of specific genotypes (11, 13). The rapid rise of M4572 to overtake M3562 suggests that M4572 gained a competitive advantage during the study period, likely driven by the formation of a distinct population immunological niche. Specifically, prolonged predominance of M3562 (P1-2) in the local population led to accumulation of P1-2-specific antibodies, while P1-1-specific antibody levels gradually waned in the absence of sustained antigenic stimulation—particularly among young children with limited prior exposure. This immunological niche allowed M4572 (P1-1) to encounter reduced type-specific antibody pressure and thereby evade host immune clearance more effectively, facilitating its clonal expansion (14, 17, 20). In addition, adaptive variations of M4572 in the post-pandemic environment, such as improved ability to spread among susceptible populations, may have further accelerated its transmission (11, 45, 46). These population-level immunity dynamics should be taken into account when considering the prevalence and transmission of MP. Notably, after NPIs, 51 MP isolates were obtained and underwent whole-genome sequencing by our group (11), which demonstrated that these two genotypes drove the resurgence of the MP via rapid clonal expansion and transmission, providing genomic evidence supporting the epidemiological observations in the present study.

The finding that SMPP was significantly more common in M3562-infected patients (20.63%) than in M4572-infected patients (13.97%) provides valuable clinical insight. Furthermore, the proportion of SMPP showed a gradual decline from 22.75% in 2022 to 6.16% in 2024. This favorable trend may be attributed to early clinical recognition, timely initiation of empirical treatment, and improved supportive care.

Additionally, the shift in dominant genotypes (from M3562 to M4572), with the latter associated with a lower risk of severe disease, may have contributed to the reduced severity burden. Although genotype testing is not yet routine in clinical practice, it could potentially serve as a prognostic tool to identify patients at higher risk of severe disease, enabling more intensive monitoring and targeted interventions.

### Strengths and limitations

This study has several strengths, including a large sample size (9,728 MPP cases), a 3-year continuous study period, and comprehensive analysis of epidemiological, resistance, and genotype data, enhancing the reliability and generalizability of the findings. However, some limitations should be acknowledged. First, the single-center design may limit the generalizability of results to other regions with different epidemiological profiles. Second, only molecular assays (PCR) were used for MP detection without antibody testing, which may have resulted in an underestimation of the positive rate. Third, the study did not investigate the molecular mechanisms underlying macrolide resistance (e.g., 23S rRNA gene mutations) or the transmission dynamics of specific genotypes. Fourth, we did not perform P1 typing on our MP samples. We will conduct both P1 typing and MLVA typing to further investigate their correlations. Fifth, genotyping failed for some specimens due to low MP-DNA load or insufficient sample volume. Previous studies have verified the correlation between bacterial load and clinical phenotype (47, 48). Nevertheless, the pathogenesis of MPP is mainly attributed to immune dysregulation rather than direct tissue injury caused by pathogens, and abnormal immune damage runs through the whole disease course from onset to aggravation into severe cases. No significant difference in pulmonary MP load was found between children with mild and severe MPP (49). Our study reliably reflects the evolutionary trend of MP MLVA genotypes from 2022 to 2024. MP load data will be incorporated in future research to further explore its correlations with MLVA genotypes and clinical severity. Finally, MP culture is technically challenging to perform in clinical practice, so macrolide resistance in our study was only judged by PCR detection of A2063G/A2064G mutations without phenotypic verification via MIC assays. Although this molecular testing method possesses high specificity, the correlation between resistance genotypes and actual functional resistance phenotypes is not absolute. This limitation may cause biased estimation of the overall macrolide resistance rate.

### Conclusions and recommendations

This study demonstrates that MP infections in children and adolescents reached an epidemic peak between late 2023 and 2024 in the Baoding area, with the number of severe cases remaining consistently low. However, two notable trends were identified concurrently: persistently high rates of macrolide resistance and a significant shift in the dominant genotypes. To address these challenges, we recommend (i) strengthening surveillance of MP epidemiology, macrolide resistance, and genotype distribution to inform timely updates to clinical guidelines; (ii) promoting the rational use of antibiotics to reduce selective pressure for resistance; (iii) considering genotype testing for severe or refractory MPP cases to guide treatment decisions; and (iv) implementing targeted prevention measures (e.g., health education, hand hygiene, and respiratory etiquette) in school-age children and adolescents to reduce transmission. Future studies should explore the molecular mechanisms underlying genotype-related severity differences and investigate the long-term impact of MRMP on clinical outcomes.

## Data Availability

The data sets presented in this study can be found in online repositories. The data are deposited in the Genome Sequence Archive (GSA) (https://ngdc.cncb.ac.cn/gsa/) repository, and the accession number is CRA043528.
